# Enlargement of the Right Testis in Child Two Years of Age

**Published:** 1879-11-20

**Authors:** Lindsay Johnson

**Affiliations:** Demonstrator of Anatomy in Southern Medical College


### ENLARGEMENT OF THE RIGHT TESTIS IN CHILD TWO YEARS OF AGE.

BY LINDSAY JOHNSON, M. D.,
Demonstrator of Anotomy in Southern Medical College.

About a month ago P. McM. came to my office for consultation regarding an enlarged condition of his little boy's right testis, he laboring under the idea that the child was "ruptured." After making an examination of the case, I was happy to inform him that his unpleasant suspicions were groundless—that his child was only affected with a subacute hyperæmic condition of the testis, resulting from a blow or slight bruise, the child exhibiting a perfect state of health otherwise.

In the outset this condition is brought about by inordinate indulgence in venery, masturbation, any urethral excitement from exposure to cold and dampness, from fatigue or mental inquietude, from intemperance, and from various constitutional causes.

Both testes may be simultaneously affected; or, as in the above case, the swelling is confined to one—with the swelling, a hydrocele is often conjoined. As a general rule the patient's general health is good, and he is seldom debarred from exercise or prevented from attending to business. A peculiarity in the disease is, that after it has existed for some length of time, there is so little pain present that the testicle may be handled quite roughly withont exciting the slightest unpleasant sensation. Indeed, the swelling frequently attains a considerable magnitude before the patient is actually aware of the condition, so painless and insidious is the approach.

In the commencement of the disease, or even after considerable swelling of the testicle has taken place, strict confinement for a week or two to the horizontal position—elevation of the scrotal sack above the pubes, and retention there by a bag or truss, the application of leeches followed up by cold saturnine solutions, or camphorated mixture, and vinegar, or the acetated liquor ammonia, together with spare diet, occasional purgation with mercury, and total avoidance of all venerial excitement and indulgence, and tapping of the hydrocele when present, will usually effect a cure in ordinary cases.

The treatment of rhe little fellow above-mentioned was simple, indeed, and resulted happily. I ordered the child to be taken home and put on a strictly milk diet for two days—at the expiration of that time, I entered the tunica vaginalis—hydrocele existing—a fact I failed to mention in the outset, and allowed the fluid to escape, which it did freely. No pain seemed to result from the operation whatever. Placing the child partly on his back, and instructing parents to keep him in this position as much as possible, I ordered ung. hydg. nitrat. et ung. belladon. partes equales to be well rubbed over the scrotum, especially on diseased side—twice daily. In a few days I had the satisfaction of seeing the little fellow entirely well, running about as though nothing ever troubled him.

